# The Radical Extent of lymphadenectomy — D2 dissection versus complete mesocolic excision of LAparoscopic Right Colectomy for right-sided colon cancer (RELARC) trial: study protocol for a randomized controlled trial

**DOI:** 10.1186/s13063-016-1710-9

**Published:** 2016-12-08

**Authors:** Jun-Yang Lu, Lai Xu, Hua-Dan Xue, Wei-Xun Zhou, Tao Xu, Hui-Zhong Qiu, Bin Wu, Guo-Le Lin, Yi Xiao

**Affiliations:** 1 Department of General Surgery, Peking Union Medical College Hospital, Chinese Academy of Medical Sciences and Peking Union Medical College, #1 Shuai Fu Yuan Street, Beijing, 100730 People’s Republic of China; 2 Department of Radiology, Peking Union Medical College Hospital, Chinese Academy of Medical Sciences and Peking Union Medical College, #1 Shuai Fu Yuan Street, Beijing, 100730 People’s Republic of China; 3 Department of Pathology, Peking Union Medical College Hospital, Chinese Academy of Medical Sciences and Peking Union Medical College, #1 Shuai Fu Yuan Street, Beijing, 100730 People’s Republic of China; 4 Department of Epidemiology and Statistics, Institute of Basic Medical Sciences, Chinese Academy of Medical Sciences/School of Basic Medicine, Peking Union Medical College, #5 Dongdan San Tiao, Beijing, 100005 People’s Republic of China

**Keywords:** Colon cancer, Laparoscopic right colectomy, Complete mesocolic excision, Oncological outcome

## Abstract

**Background:**

The extent of lymphadenectomy during laparoscopic right colectomy can affect the oncological outcome and the safety of surgery. The principle of complete mesocolic excision (CME) has been gradually accepted and increasingly applied by colorectal surgeons. The aim of this study is to investigate whether extended lymphadenectomy (CME) in laparoscopic colectomy could improve the oncological outcomes of patients with right-sided colon cancers, compared with D2 lymphadenectomy.

**Methods/design:**

The Radical Extent of lympadenectomy: D2 dissection versus complete mesocolic excision of LAparoscopic Right Colectomy for right-sided colon cancer (RELARC) study is a prospective, multicenter, randomized controlled trial in which 1072 eligible patients with right-sided colon cancers will be randomly assigned to the CME group or the D2 dissection group during laparoscopic right colectomy. Inclusion criteria are locally advanced colon cancers situated from the cecum to the right third of the transverse colon and clinically staged as T2-4aN0M0 or TanyN + M0. The primary endpoint of this trial is 3-year disease-free survival. Secondary endpoints include 3-year overall survival, postoperative complication rates, perioperative mortality rates, and rates of positive central lymph nodes (the station 3 nodes).

**Discussion:**

The RELARC trial is a prospective, multicenter, randomized controlled trial that will provide evidence on the optimal extent of lymphadenectomy during laparoscopic right colectomy in terms of better oncological outcome and operation safety.

**Trial registration:**

ClinicalTrials.gov: NCT02619942. Registered on 29 November 2015.

**Electronic supplementary material:**

The online version of this article (doi:10.1186/s13063-016-1710-9) contains supplementary material, which is available to authorized users.

## Background

Colorectal cancer is one of the most common malignant diseases worldwide. In China, both the incidence and mortality of the disease have been increasing during the past decades, due to the aging population, changing lifestyles, and eating habits. In 2011, an estimated incidence of 23.03/10 million was reported in China, accounting for the fourth most common type of malignant tumor. With a mortality rate of approximately 11.11/10 million, colorectal cancer was the fifth leading cause of cancer-related death in China [1].

Since Hohenberger first proposed the concept of complete mesocolic excision (CME) in 2009, the principle of CME has been gradually accepted and increasingly applied by colorectal surgeons as an optimal colectomy approach for colon cancer [2, 3]. According to this principle, the extent of lymphadenectomy of the radical right colectomy is similar to that of D3 lymph node dissection, which was advocated by the Japanese Society for Cancer of the Colon and Rectum (JSCCR) in the twentieth century [4]. In CME, the central vascular ligation (CVL) enables more radical removal of the metastatic lymph nodes. The resection in the mesocolic plane guarantees radical excision of the tumor and the potential vascular or neural infiltration in the whole regional draining area, which theoretically would improve the oncological outcome in comparison with the conventional right colectomy with D2 lymphadenectomy. However, compared to D2 dissection, laparoscopic CME or D3 dissection imposes a longer learning curve for the surgeons and a higher surgery-related risk for patients due to the complicated surgical anatomy involved in the laparoscopic approach, especially the dissection in the vicinity of the superior mesenteric vessels [5–8]. Neither the National Comprehensive Cancer Network (NCCN) guidelines nor the European Society for Medical Oncology (ESMO) guidelines have clearly defined the extent of lymphadenectomy of the radical right colectomy [9]. D3 lymphadenectomy was also advocated for locally advanced colorectal cancer by the Chinese standard for diagnosis and treatment of colorectal cancer, but CME was not mentioned [10]. In addition, all the results indicating improved survival rate of CME surgery compared to traditional D2 dissection were drawn from retrospective studies [2, 11–15]. Therefore, a well-designed prospective clinical trial is crucial to investigate the optimal extent of lymphadenectomy of laparoscopic right colectomy for right-sided colon cancer. The study protocol has been written in accordance with the Standard Protocol Items: Recommendations for Interventional Trials (SPIRIT) checklist (see Additional file 1).

## Methods/design

### Study design

This study is a superiority trial. The main objective is to investigate whether extended lymphadenectomy (CME) in laparoscopic colectomy could improve the oncological outcomes of patients with right-sided colon cancer, compared to laparoscopic colectomy with D2 lymphadenectomy.

The study design is a prospective, multicenter, randomized, two-arm, parallel-group, single-blind clinical trial. The enrolled participants with right-sided colon cancers, including cancers situated from the cecum to the right third of the transverse colon, will be divided into two groups: the laparoscopic CME group (the intervention group) and the laparoscopic colectomy with D2 dissection group (the control group).

According to the final pathological results of surgical specimens, patients with stage III colon cancer or stage II disease with unfavorable histological features will be required to undergo adjuvant chemotherapy for 6 months. The oxaliplatin-based combination regimen will be recommended.

All the patients will be followed up regularly for at least 5 years, according to the follow-up protocol recommended by the NCCN guidelines. All patients are required to undergo postoperative examinations every 4 months for the first 2 years and every 6 months thereafter until 5 years after the surgery. Signs of suspected disease relapse including local recurrence (LR) or distant metastasis (DM) will be closely observed and monitored. The schedule of this trial is shown in Table 1.

**Table 1:** Schedule of enrollment, intervention, and assessments for the RELARC trial

	Study period										
	Enrollment	Allocation	Post-allocation								Close-out
Timepoint	–2 weeks	0	Surgery	4 months	8 months	1 year	16 months	20 months	2 years	30 months	3 years
Enrollment:											
Eligibility screen	X										
Informed consent	X										
Allocation		X									
Interventions:											
Laparoscopic D2 operation			X								
Laparoscopic CME operation			X								
Assessments:											
Disease-free survival				X	X	X	X	X	X	X	X
Overall survival				X	X	X	X	X	X	X	X
Postoperative complication			X								
Perioperative mortality			X								
Central lymph node assessment			X								

Our study is expected to last 7 years, including 4 years for recruiting patients and 3 years for follow-up.

### Endpoints

The primary endpoint of this trial is 3-year disease-free survival (DFS). Secondary endpoints include 3-year overall survival (OS), postoperative complication rates, perioperative mortality rates, and rates of positive central lymph nodes (the station 3 nodes).

#### Definition of some postoperative complications

Anastomotic leak, postoperative diarrhea, and chylous ascites are possible postoperative complications. Anastomotic leak is described as the presence of luminal contents through a drain or wound site or an abscess cavity causing inflammation (i.e., fever, leukocytosis, or fecal discharge). An anastomotic leak may be detected using radiologic studies, but it must exhibit clinical signs of leakage to be considered a clinical leak.

Postoperative diarrhea shows abnormal feces, such as loose stool, watery stool, mucous stool, or bloody purulent stool three times or more a day within 2 weeks after the operation.

Chylous ascites is defined as the presence of non-infectious milky or creamy peritoneal fluid in the drainage tubes with a volume of ≥200 mL/day and a positive chyle test.

### Participating centers and investigators

Seventeen tertiary university hospitals will enroll patients in China. The surgeons who participate in the study should have experience of more than 100 cases of laparoscopic colorectal surgery per year for 2 consecutive years and the ability to complete the skilled laparoscopic CME procedure. Every investigator should provide operation videos of CME and D2 procedures within the past 3 months; these videos will be reviewed by the review committee.

### Ethical considerations

This trial protocol is approved by the Ethics Committee of Peking Union Medical College Hospital (reviewed in 2015 as ZS-851) and has been registered at ClinicalTrials.gov (identifier NCT02619942). All of the eligible participants and their legal surrogates will be fully informed of the potential risks and benefits of the interventions in each group. Only patients who are willing to provide written informed consent can be enrolled in the trial.

### Study subjects

The study subjects are patients with locally advanced right-sided colon cancers, including carcinomas located at the cecum, the ascending colon, the hepatic flexure, and the right third of the transverse colon.

The inclusion criteria are as follows:

1. Age 18–75 years old
2. Pathologically confirmed adenocarcinoma of right side of colon
3. Localization of the tumor ranging from the cecum to the right third of the transverse colon
4. Tumors clinically staged as T2-4aN0M0 or TanyN + M0, according to imaging studies including enhanced computed tomography (CT) scan, magnetic resonance imaging (MRI), etc.
5. American Society of Anesthesiologists (ASA) score of I, II, or III.

The exclusion criteria are as follows:

1. Synchronous or metachronous multiple primary colorectal cancer
2. Preoperative imaging examination shows enlargement of the central lymph nodes, which mandates a D3 lymphadenectomy
3. Preoperative imaging examination shows that the tumor involves the adjacent organs, requiring combined multiple organ resections, or radical excision (R0 resection) is not possible
4. History of any other malignant tumor in past 5 years, except for cervical carcinoma in situ that has been cured, basal cell carcinoma, or squamous cell carcinoma of skin
5. Patients who need an emergency operation
6. Patients who are not suitable to undergo laparoscopic surgery (due to, e.g., extensive adhesion caused by prior abdominal surgery, inability to endure artificial pneumoperitoneum, etc.).

Exit criteria include:

1. Patients with distant metastasis that is discovered during surgical exploration or confirmed by the postoperative pathologic examination
2. Combined organ resection during the surgery
3. Fusion of central lymph nodes at the mesenteric root discovered during the operation.

This study is scheduled to enroll consecutive patients in 17 tertiary medical centers in China. Patients enrolled in this clinical research will be guaranteed high-quality surgical procedures and standard medical treatment as well as convenient long-term follow-up.

### Randomization and blinding

Patients enrolled in this study will be randomly assigned into either the study group or the control group. The random numbers are generated using Statistical Analysis System (SAS) software to ensure that patients are randomly divided into two groups with an allocation ratio of 1:1. A central randomization system will be employed, and all the researchers must register at the website of the research system. When a researcher has chosen a certain patient, he/she should log on to the webpage and upload the basic information on the patient before acquiring the random grouping schemes.

All the patients are blinded to the surgical approach that they are going to receive, and will be required to sign the informed consent before being enrolled in the study. Obviously, it is impossible to blind the surgeons who will perform the surgeries. However, all the surgeon researchers will be required to limit potential co-interventions as much as possible. In addition, the result assessment will be made by independent researchers who are blinded to the surgical procedures.

### Preoperative evaluation

A diagnosis of colon cancer must be pathologically confirmed before the surgery, using the biopsy specimen acquired from the colonoscopy. An enhanced CT scan of the abdominal and pelvic cavity is performed to localize the lesion. A CT colonography (virtual colonoscopy) or double-contrast barium enema can be performed when necessary.

An enhanced CT scan of the abdomen and pelvis can also provide information about the depth of tumor infiltration, the status of the regional lymph node metastasis, and the involvement of adjacent organs. A whole body enhanced CT scan can also demonstrate distant metastatic disease, such as liver, lung, and even peritoneum metastases. If necessary, other examinations including abdominopelvic MRI, contrast-enhanced ultrasound of the liver, endoscopic ultrasound, whole body bone scan, and positron emission tomography (PET)-CT can be used to facilitate the preoperative evaluation.

### Surgery

Surgical procedures for the two groups require sharp dissection strictly following the anatomical planes (also called embryonic planes) [2], including both the anterior and posterior aspects of the mesocolon, and separate the Toldt’s fascia from the retroperitoneum with an intact package of the tumor and its main lymphatic drainage (see Additional file 2). Complete mobilization of the mesocolon and complete removal of the vessels and lymph nodes inside will be achieved. The main differences between the two groups include the extent of lymphadenectomy (D2 versus D3); and whether or not to dissect the lymphoadipose tissues at the root of the mesocolon, which cover the anterior surface of the superior mesenteric artery (SMA) and vein (SMV), and the tissues surrounding the root of the middle colic artery.

There is no difference between the two groups in the extent of longitudinal resection. All procedures are basically a right hemicolectomy. The ileum is divided at 5–10 cm of the cecum. For cancer of the cecum and ascending colon, only the right branches of the middle colic vessels are divided centrally, and the colon is divided right at the level of the middle colic vessels. Cancer of the hepatic flexure and the right third of the transverse colon requires true central ligation of the middle colic artery and vein with resection of the transverse colon in the proximity of the splenic flexure, as well as a central tie of the right gastroepiploic artery and vein.

### For D2 lymphadenectomy

**Fig. 1:**
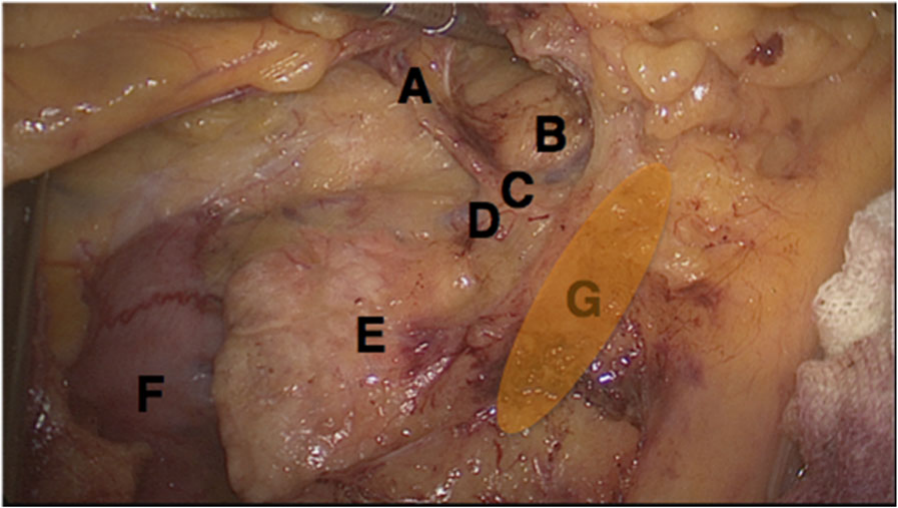
D2 lymphadenectomy requires dissecting the supplying vessels right of the superior mesenteric vein (*SMV*) and keeping the covering lymphoadipose tissue of the SMV intact. **a** Right colic vein. **b** Pancreas neck. **c** Henle’s trunk. **d** Inferior pancreaticoduodenal vein. **e** Pancreas head. **f** Duodenum. **g** Undissected SMV

**Fig. 2:**
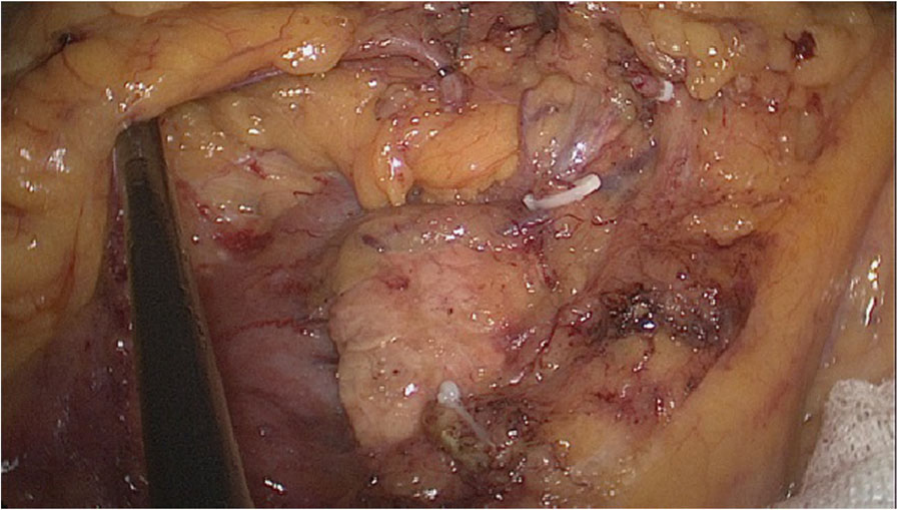
During D2 lymphadenectomy, the supplying vessels are ligated right of the SMV

The D2 lymphadenectomy is performed to remove the whole anterior and posterior aspects of the mesocolon and the lymphoadipose tissues covering the anterior surface of the pancreatic head and neck, which represent the mesenteric roots of the ascending colon and the right third of the transverse colon respectively (Fig. 1). This procedure is required along the anatomical planes (embryonic planes) and is followed by ligation of the colonic supplying vessels right of the SMV and cleaning up of the surrounding lymphoadipose tissue (Fig. 2).

### For CME procedure

**Fig. 3:**
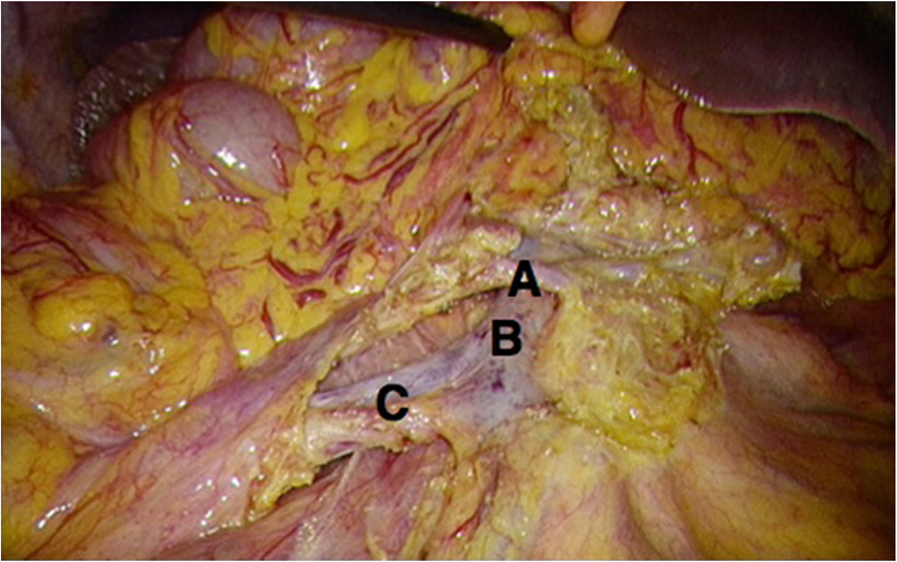
The CME procedure requires cleaning the lymphoadipose tissue on the surface of the SMA and ligating the supplying vessels at their origins. **a** Right colic artery. **b** Superior mesenteric vein. **c** Ileocecal artery and vein

**Fig. 4:**
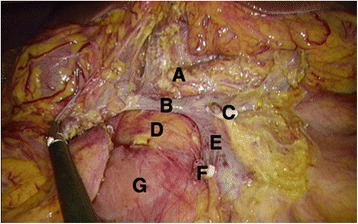
After complete removal of the ascending and transverse mesocolon from the roots, the pancreatic head and neck are clearly exposed during the CME procedure. **a** Pancreas neck. **b** Henle’s trunk. **c** Ligated right colic artery. **d** Pancreas head. **e** Superior mesenteric vein (surgical trunk). **f** Ligated ileocecal vessels. **g** Duodenum

In addition to the dissecting area described in the D2 procedure, the CME procedure cleans up the lymphoadipose tissue on the surface of the SMA and SMV. This requires further exposure and skeletalization of the right and anterior sides of the SMV, including the surgical trunk. This exposure should remove the lymphoadipose tissues instead of the connective tissues covering the SMA, to expose its outline (Fig. 3). Then, it is followed by the ligation of the supplying vessels (both artery and vein) at their origins; the results are shown in Fig. 4.

### Postoperative evaluation

At the end of the surgery, the surgeon must take photos of the specimens and upload them to the central website. Both the anterior and posterior sides of the specimens are taken alongside a metric scale for calibration. The mesentery is laid out flat without external tension, indicating the sites of the tumor and vascular ties. The diameter of the tumor, the distance between the proximal and distal cutting edge to the tumor, and the distance between the most distant free mesocolon and the tumor are also precisely measured. The area of mesentery resected will be calculated after the operation by the parameters collected in the operation room.

The quality of the surgical specimens is graded from the integrity of the mesocolon by an independent review committee using a method described previously [3]. The grading of surgical specimens is classified into three groups: good (intact anterior and posterior fascia of mesocolon, intact lymphoadipose tissue from the surface of SMV in CME procedure), moderate (defects of mesocolic fascia that expose the mesocolic fat, but the incisions do not reach down to the muscularis propria), or poor (significant defects of mesocolon or disruptions extending down onto the muscularis propria). Any discrepancies of the grades will be discussed before a final grade is agreed on.

### Postoperative adjuvant therapy

The postoperative adjuvant chemotherapy is determined by the pathological results. For patients with stage III disease and patients of stage II with unfavorable histological features, 6 months of adjuvant chemotherapy using an oxaliplatin-based combination regimen is recommended. For patients of stage II without unfavorable histological features, adjuvant chemotherapy is determined by the microsatellite instability (MSI) state. For patients with microsatellite stability (MSS), single-agent chemotherapy based on 5-fluorouracil (5-FU) is recommended. For patients with MSI, we suggest just observation and regular follow-ups.

### Follow-up and data collection

The postoperative examination will be performed every 4 months in the first 2 years and every 6 months in the following 3 years. Disease-free survival (DFS) is defined as the time from randomization to the time of finding the recurrence, metastasis, or death. It is calculated on a monthly basis for the latest finding. Local recurrence is defined as a recurrence of the tumor in the surgical region, with or without elevation of serum tumor markers, and the mass of the anastomotic site must be confirmed by a pathological biopsy. A distant metastasis is defined as a metastasis that is discovered in the liver, lung, bone, or other sites by CT, MRI, or radionuclide scanning, with or without pathological examination. The postoperative complications that are included in the statistics include intra-abdominal hemorrhage, gastrointestinal bleeding, anastomotic leakage, chylous fistula, surgical site infection, including intra-abdominal infections and wound infection, intestinal obstruction, postoperative diarrhea, pulmonary infection, urinary tract infection, cardiovascular accident, cerebral vascular accident, and thrombotic disease.

### Sample size calculation

Previous retrospective studies reported that the 3-year DFS for the D2 operation ranged from 66.7% to 82.1%; the range was 77.4% to 89.1% for the CME operation [10, 15]. We estimate that the 3-year DFS for D2 and CME will be 72.0% and 80.0% respectively in this study. We expect to detect a difference of 8% in 3-year DFS between the two groups. Thus, with a significance level of 5%, a two-sided test, and 80% power, 447 patients will be required for each group. With a drop-out rate of 20%, the required sample size is 1072 patients for both arms.

### Statistical analysis

Continuous variables will be expressed as mean, standard deviation, median, minimum, maximum, Q1, and Q3. Categorical variables will be expressed as numbers and percentages. The *t* test and Wilcoxon rank sum test will be used to compare continuous variables, and chi-square analysis/Fisher’s exact test will be used to compare categorical variables. Survival analysis will be conducted using the Kaplan-Meier method and the log-rank test. Statistical analysis will be carried out using SAS 9.2. All statistical tests are two-sided tests, and a *P* value less than or equal to 0.05 is considered as statistically significant.

### Data and safety monitoring

An independent Data and Safety Monitoring Board (DSMB) including senior colorectal surgeons, biostatisticians, a research contractor, and an ethicist has been set up. DSMB members will meet before the study and every 6 months during the study. The DSMB will review elements of the study, including adverse events (AEs), drop-outs, and endpoints. If the interim results show a huge difference between the two groups that is much more than anticipated, the DSMB can stop the study early for the safety and benefit of the patients.

## Discussion

In the past 30 years, the development of surgery for colorectal cancer has been unbalanced. The surgical procedure for colon cancer is relatively unchanged, but the surgical treatment of rectal cancer has been constantly evolving. Contributing factors to this situation include the double-stapling technique, the principle of total mesorectal excision (TME), the 2-cm distal margin, neoadjuvant therapy, and other new technologies.

The transformation of traditional open surgery to minimally invasive surgery has become an inevitable trend. Some high-level clinical studies have shown that laparoscopic surgery and open surgery did not have significant differences in the long-term oncological outcomes or the short-term effect for colorectal cancer [16–18]. The 2015 NCCN guidelines for colon cancer advocated laparoscopic colectomy to experienced surgeons. The incidence of right colon cancer accounts for more than 50% of colon cancer occurrence. Right colectomy is relatively consistent, and open right colectomy is even considered to be the entry procedure for colorectal specialists. The anatomical layers in laparoscopic right colectomy, however, are more complicated compared to other parts of colon cancer surgery by the laparoscopic approach, and vascular variation in this region is common. Therefore, the difficulty of laparoscopic right colectomy is greater than that of the open procedure [5]. Neither the NCCN nor the ESMO guidelines have clearly defined the extent of lymphadenectomy for a radical right colectomy. Therefore, the key steps of laparoscopic right colectomy are still controversial.

The concepts of D3 dissection and CME developed in different eras and different countries. The concept of CME historically originated from TME, which emphasized dissection along the embryologic plane and ligation of the supplying vessels at their origins. The other difference between D3 dissection and CME is the longitudinal resection (longitudinal margin of 10 cm versus right hemicolectomy), the latter technique bring a more extensive longitudinal removal. In the present study, all procedures require sharp dissection strictly following the embryologic plane and are basically a right hemicolectomy, which is different from D3 dissection. For cancer of the hepatic flexure and right third of the transverse colon, more extensive resection of the transverse colon in the proximity of the splenic flexure is recommended.

In the past 30 years, similar disputes or innovations for gastrointestinal surgeries include the principle of TME for rectal cancer, the extent of lymph node dissection for gastric cancer, and lateral lymph node dissection for rectal cancer, etc. The principle of TME received general approval immediately after it came out because it did not increase the difficulty of the operation and achieved less bleeding, although it was not confirmed by randomized controlled studies. However, other issues, including D2 or more extended lymphadenectomy for advanced gastric cancer [19, 20] and the necessity of lateral lymph node dissection for rectal cancer, were controversial [21]. Eventually, some advanced studies of evidence-based medicine settled the arguments and formed the current guidelines. The concept of CME originated from TME, but whether it can achieve the same acceptance as TME still needs to be verified in practice.

### Trial status

This trial was initiated in January 2016 and is currently recruiting patients.

## Additional files

Additional file 1: SPIRIT 2013 checklist. (DOCX 26 kb)
Additional file 2: Schematic diagram depicting complete mobilization of mesocolon. The plane of dissection lies between Toldt’s fascia and the retroperitoneum. (JPG 823 kb)

## Abbreviations

AE: Adverse event
ASA: American Society of Anesthesiologists
CME: Complete mesocolic excision
CT: Computed tomography
CVL: Central vascular ligation
DFS: Disease-free survival
DM: Distant metastasis
DSMB: Data and Safety Monitoring Board
ESMO: European Society for Medical Oncology
FU: Fluorouracil
JSCCR: Japanese Society for Cancer of the Colon and Rectum
LR: Local recurrence
MRI: Magnetic resonance imaging
NCCN: National Comprehensive Cancer Network
OS: Overall survival
RELARC: Radical Extent of lymphadenectomy of LAparoscopic Right Colectomy for right-sided colon cancer
SMA: Superior mesenteric artery
SMV: Superior mesenteric vein
TME: Total mesorectal excision
