# Supplementary figures and images for: The Radical Extent of lymphadenectomy — D2 dissection versus complete mesocolic excision of LAparoscopic Right Colectomy for right-sided colon cancer (RELARC) trial: study protocol for a randomized controlled trial
Source: Trials. 2016 Dec 8;17:582. doi: 10.1186/s13063-016-1710-9 (PMC5146822; doi:10.1186/s13063-016-1710-9)

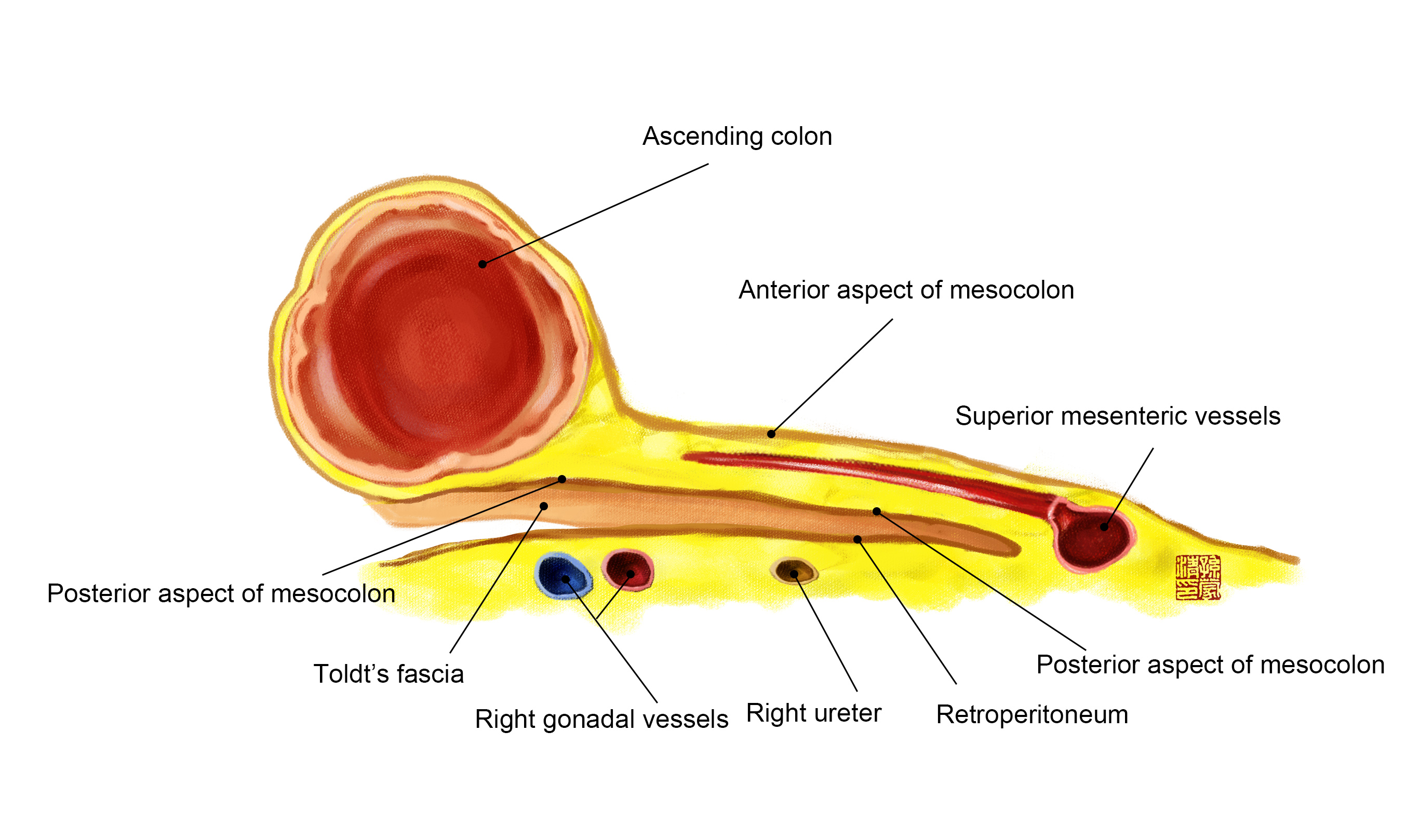

Supplement: Additional file 2: — Schematic diagram depicting complete mobilization of mesocolon. The plane of dissection lies between Toldt’s fascia and the retroperitoneum. (JPG 823 kb) [file 13063_2016_1710_MOESM2_ESM.jpg]
